# NCBO Technology: Powering semantically aware applications

**DOI:** 10.1186/2041-1480-4-S1-S8

**Published:** 2013-04-15

**Authors:** Patricia L Whetzel, NCBO Team

**Affiliations:** 1 Stanford Center for Biomedical Informatics Research, Stanford University, Stanford, CA 94305, USA; 2 Division of Biomedical Statistics and Informatics, Mayo Clinic, Rochester, Minnesota, USA; 3 Department of Computer Science, University of Victoria, Victoria, British Columbia, Canada; 4 Department of Philosophy, University at Buffalo, Buffalo, New York, USA

**Keywords:** BioPortal, ontology, web service, REST, Annotator, Resource Index

## Abstract

As new biomedical technologies are developed, the amount of publically available biomedical data continues to increase. To help manage these vast and disparate data sources, researchers have turned to the Semantic Web. Specifically, ontologies are used in data annotation, natural language processing, information retrieval, clinical decision support, and data integration tasks. The development of software applications to perform these tasks requires the integration of Web services to incorporate the wide variety of ontologies used in the health care and life sciences. The National Center for Biomedical Ontology, a National Center for Biomedical Computing created under the NIH Roadmap, developed BioPortal, which provides access to one of the largest repositories of biomedical ontologies. The NCBO Web services provide programmtic access to these ontologies and can be grouped into four categories; *Ontology*, *Mapping*, *Annotation*, and *Data Access.* The *Ontology* Web services provide access to ontologies, their metadata, ontology versions, downloads, navigation of the class hierarchy (parents, children, siblings) and details of each term. The *Mapping* Web services provide access to the millions of ontology mappings published in BioPortal. The *NCBO Annotator* Web service “tags” text automatically with terms from ontologies in BioPortal, and the *NCBO Resource Ind*ex Web services provides access to an ontology-based index of public, online data resources. The NCBO Widgets package the *Ontology* Web services for use directly in Web sites. The functionality of the NCBO Web services and widgets are incorporated into semantically aware applications for ontology development and visualization, data annotation, and data integration. This overview will describe these classes of applications, discuss a few examples of each type, and which NCBO Web services are used by these applications.

## NCBO Technology overview

BioPortal is an open repository of biomedical ontologies that stores ontologies developed in various formats, such as OWL, OBO format, Protégé frames, and the Rich release format, and provides access to this content via Web browsers and Web services [1,2]. The BioPortal Web interface allows users to browse the list of ontologies, search and comment on terms in ontologies, annotate text with ontology terms, and search an ontology-based index of biomedical resources. The BioPortal architecture currently includes both LexEVS (http://informatics.mayo.edu/LexGrid) and the Protégé database (http://protege.stanford.edu), however work is underway to replace the dual database backend with a RDF database. A beta version of the BioPortal RDF database is available at: http://sparql.bioontology.org.

**Figure 1:**
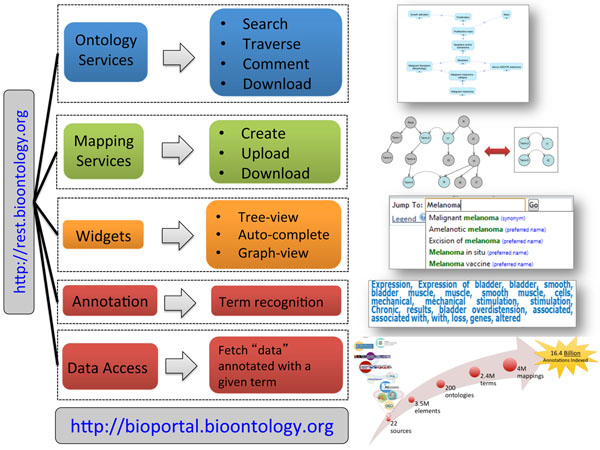
**NCBO Technology** The NCBO Web services and widgets provide access to ontologies in BioPortal. The Web services can be grouped into four categories; Ontology, Mapping, Annotation and Data Access.

The functionality of the BioPortal Web site is driven by the NCBO Web services, which include the *Ontology*, *Mapping*, *Annotator*, and *Resource Index* Web services (Figure 1). The *Ontology* Web services provide access to ontologies, their metadata, ontology versions, navigation of the class hierarchy (parents, children, siblings) and details of each term. These services also allow download of the ontology (in the original format and in RDF), provide the ability to search for terms in an ontology, to extract subsets of an ontology and to provide comments and propose new terms as metadata to the ontology. The *Widgets* package the functionality of the *Ontology* Web services in order to provide embeddable code for Web sites. These widgets include a term autocomplete widget and ontology visualization widgets. The *Mapping* Web services provides access to a variety of mappings published in BioPortal. The mapping data includes mappings from UMLS based on shared Concept Unique Identifiers, mappings specified within ontologies, user submitted mappings, and automatically generated mappings using the Lexical OWL Ontology Matcher (LOOM), which generates mappings based on lexical similarity of the preferred name and synonyms between pairs of ontologies [3]. The *Mapping* Web services are parameterized to allow a high degree of flexibility to access the data. For example, mappings can be accessed for one ontology mapped to all other ontologies, between pairs of ontologies, for one term mapped to all other terms, and between pairs of terms. This Web service can also be used to submit mappings directly to BioPortal. The *NCBO Annotator* Web service processes text to recognize terms from ontologies in BioPortal that exist within the text [4]. The *Annotator* Web service uses the entity recognizer Mgrep [5], which outperforms MetaMap in almost all cases evaluated for precision [6]. The Web service parameters can be set to limit results to a particular ontology or to certain UMLS semantic types and characterisitcs of the term matches can also be parameterized, e.g. to recognize both preferred name and synonyms, match terms greater than X characters in length, and the ability to include a custom list of stopwords. The *NCBO Annotator* Web service was used to generate an ontology-based index of several online biomedical data repositories (e.g., GEO, ClinicalTrials.gov, dbGaP, DrugBank, PharmGKB, and Reactome) resulting in the *NCBO Resource Index *[7,8]. The textual metadata of data records from these resources was annotated with terms from ontologies in BioPortal and then stored locally for query efficiency. Therefore, data records across databases are linked together via their shared ontology annotations. These linkages take advantage of the semantic relationships within the ontology, including subsumption relationships among ontology entities and mappings between entities in different ontologies. The *Resource Index* is designed to *provide* updates in both new resource data records and new ontology versions. The *NCBO Resource Index* Web services provide a mechanism for programmatic search of the index using ontology terms. For example, one can search for all experiments and clinical trials related to ‘malignant melanoma’ from GEO and ClinicalTrials.gov. The NCBO Web services are documented at: http://www.bioontology.org/wiki/index.php/NCBO_REST_services

## Classes of applications incorporating ontologies via NCBO Technology

### Ontology development and visualization

With the growing interest in the use of ontologies in the health care and life sciences, additional tools are being developed to support the development of ontologies within new biomedical domains and the re-use of existing ontologies to build application ontologies. To this end, new plugins for ontology editing tools such as Protégé and OBO-Edit have been developed. These plugins use the NCBO Web services to aid in term re-use, to automatically generate ontology terms from text, provide an infrastructure for collaborative ontology development, and provide ways to visualize ontologies.

**Figure 2:**
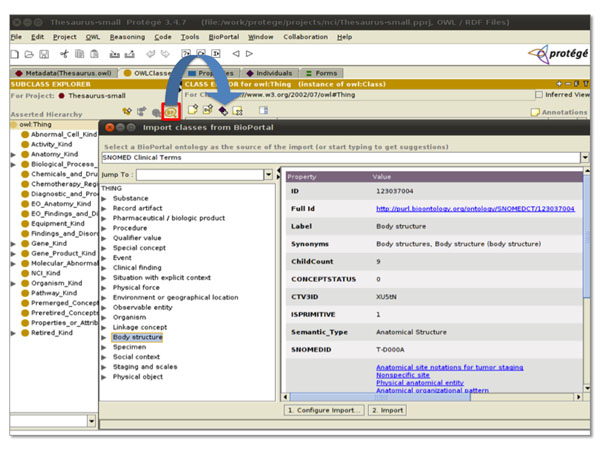
**BioPortal Import plugin for Protégé** The BioPortal Import plugin allows ontology developers to search for terms within ontologies in BioPortal and import these into their own ontology directly from Protégé.

The ***BioPortal Import plugin ***[9] enables re-use of ontology terms by allowing the ontology developer to search for terms in BioPortal directly from Protégé 3 (Figure 2). The terms of interest can be directly imported enabling the re-use of terms rather than creating new terms with new URIs. The developer can import an entire subtree of terms and specify the desired depth of child terms to import. The annotation properties of the imported terms can be specified in order to harmonize these properties with existing terms in the new application ontology. The ***BioPortal Reference plugin ***[10] also enables term re-use, however in this case by generating references to external ontologies stored in BioPortal. This method is being used in the development of the International Classification of Diseases, version 11 and minimizes the size of the ontology via the reference without sacrificing content. These plugins are powered by *Ontology* Web services including the “List all Ontologies”, “Search”, “Get Term”, and “View Extraction” Web services.

The ***OLS2OWL plugin ***[11] is designed to aid ontology developers during the knowledge elicitation stage and allows ontology developers to search for terms from a repository of ontologies and compare similar classes, properties, and instances. The plugin was developed as part of the Open architecture for Accessible Services Integration and Standardization project, which facilitates interoperability across service providers, mobile devices (wearable devices, phones, palm, etc.) smart home technology, and medical care providers for elderly and disabled population. The ***Dresden Ontology Generator for Directed Acyclic Graphs ***[12] plugin for Protégé 4 and OBO-Edit generates ontology terms, definitions, and relationships based on natural language text found in PubMed, the Web, or PDF documents and therefore supports the extension of existing ontologies with terms from resources commonly used in biocuration. These tools use the “List all Ontologies”, “Search”, and “Get Term” Web services.

In addition to tools for ontology re-use, infrastructure now exists for collaborative ontology development, a methodology commonly used in biomedical ontology development. ***WebProtégé ***[13] is a web-based ontology-editing environment, which supports collaboration, enabling users to edit an ontology simultaneously, carry out discussions, and add comments to the terms. These comments and new term proposals can be submitted and viewed in BioPortal using the “Notes” Web services.

**Figure 3:**
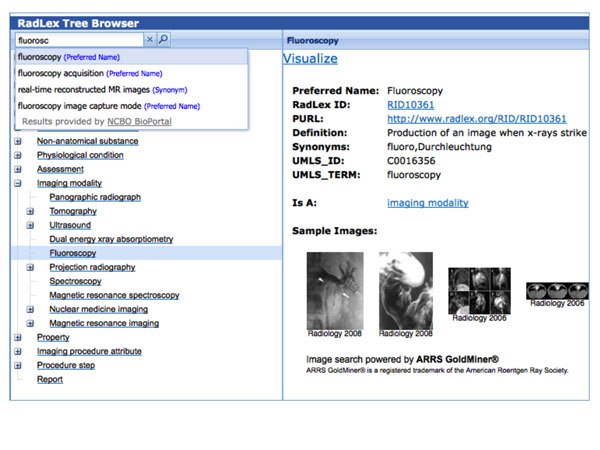
**RadLex Term Browser** RadLex term details and hierarchy are displayed using the NCBO Web services. The browser is customized to display additional term details, e.g. Sample Images, and the user interface is designed to meet the requirements of the Radiological Society of North America.

The display of an ontology, i.e. the tree hierarchy and term details, can also be customized for display in term browsers. For example, the ***RadLex Term Browser ***[14] uses the “Hierarchy” Web services to display the ontology tree and “Get Term” Web service to display the term details formatted and customized to meet the requirements of the Radiological Society of North America (Figure 3). The browser also uses the “Notes” Web service to allow users to submit new term proposals for review and inclusion in the ontology.

### Data annotation

Ontologies are also commonly incorporated into data annotation applications. While BioPortal contains over 400 ontologies, to help identify ontologies that best cover the text for annotation the *Ontology Recommender* Web service can be used. The input to this Web service is either a list of terms or corpus of text and generates a ranked list of what ontologies best cover the text. The resulting ontologies can then be selected for use in data annotation applications and terms presented to the user in various ways. Data annotation applications represent the most widely used category of applications using the NCBO Web services.

**Figure 4:**
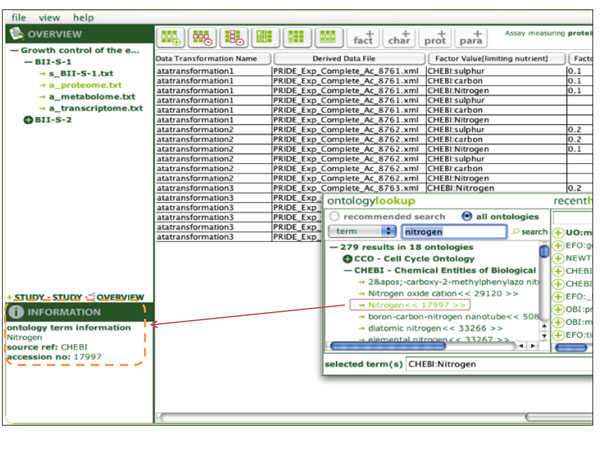
**ISACreator** ISACreator allows data annotators to dynamically select ontology terms for use in annotation tasks.

For example, the ***ISAcreator ***[15] tool is configured to use specific ontologies (Figure 4). For data fields requiring an ontology term, the user can search for these terms from the ISAcreator application using the “Search” Web service. These fatures are also included within ***OntoMaton ***[16] available from the Google Script Gallery. ***Rightfield ***[17] also constrains data annotation to certain ontologies via an Excel spreadsheet. A Web-based application is used to configure the ontology selection and data input fields. The “Ontology Download” Web service is then used together with the configuration to generate an Excel form that can be populated as a desktop application.

**Figure 5:**
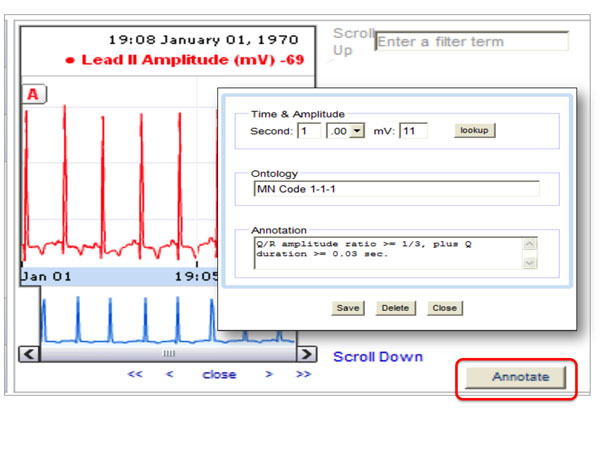
**ECG Gadget** The ECG Gadget displays electronic ECG traces and provides a mechanism for physicians to annotate both waves and intervals in the ECG trace.

The ***ECG Gadget ***[18] is a tool developed by the CardioVascular Research Grid that enables physicians to annotate electronic ECG traces (Figure 5). The tool is developed using the Google Web Toolkit and uses the “Search” and “Get Term” Web services to access terms of interest and display the content such as the term definition so that the annotator can confirm the selection of the correct term.

The NCBO *Ontology* Web services are also used in applications to harmonize data elements. For example, ***openMDR ***[19] uses the “List all Ontologies”, “Search”, and “Get Term” Web services to provide access for curators to select terms from ontologies such as the NCI Thesaurus, Ontology for Clinical Research, or SNOMED-CT. ***eleMap ***[20], a tool developed by the eMERGE Network [21], follows a similar workflow. The tool provides a mechanism for researchers to harmonize their local phenotype data dictionaries to existing metadata and terminology standards such as the Cancer Data Standards Registry and Repository, the NCI Thesaurus, and SNOMED-CT.

**Figure 6:**
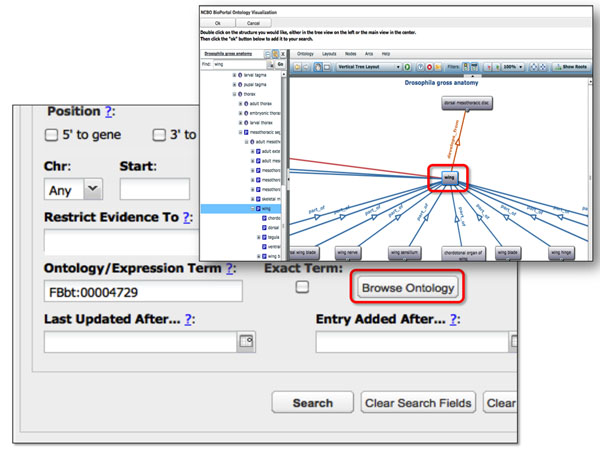
**REDfly** REDfly is a data annotation tool and enables annotators to view the ontology hierarchy and graph from within the annotation tool. Once a term is selected it is automatically populated into the data annotation tool.

The Web services are also provided as Web widgets to ease application development. For example, the “Search” Web service drives and Term auto-complete widget, while the “Hierarchy” and “Get Term” Web services drive the graph and tree visualization widgets. These widgets are also used in data annotation applications such as ***RedFly ***[22] (Figure 6) and the ***Knowledge Egg ***[23].

### Data integration

While many projects aim to collect annotated data upon submission of new data sets, unstructured text also accompanies data sets. The *Annotator* Web service can be used in these cases to identify ontology terms within a corpus of text and the data sets can be linked via these ontology annotations.

**Figure 7:**
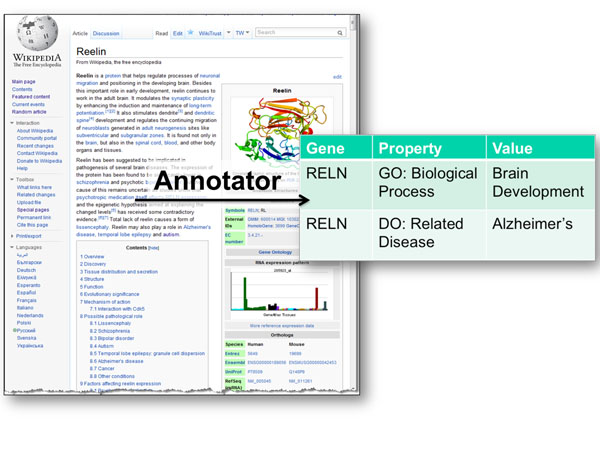
**GeneWiki** Text from the GeneWiki article, in this example for the glycoprotein Reelin, is used as input to the *Annotator* Web service to identify ontology terms from the Gene Ontology and Disease Ontology.

The ***GeneWiki ***[24] contains information about human genes and seeks to apply community intelligence to the annotation of gene and protein function (Figure 7). To identify mentions of disease and biological processes within GeneWiki articles, the *Annotator* Web service is used to identify terms from the Gene Ontology and Disease Ontology.

The ***NCBO Resource Index*** is an ontology-based index of publicly available biomedical databases. The text descriptions of database entries are processed using the *Annotator* Web service to identify ontology terms and then the results are stored in the Resource Index. The ontology-based index links the data records within a database and across disparate databases, providing a functional linkage based on the content of the data field as opposed to schema matching. These annotations and linkages are useful to more precisely identify data records of interest.

**Figure 8:**
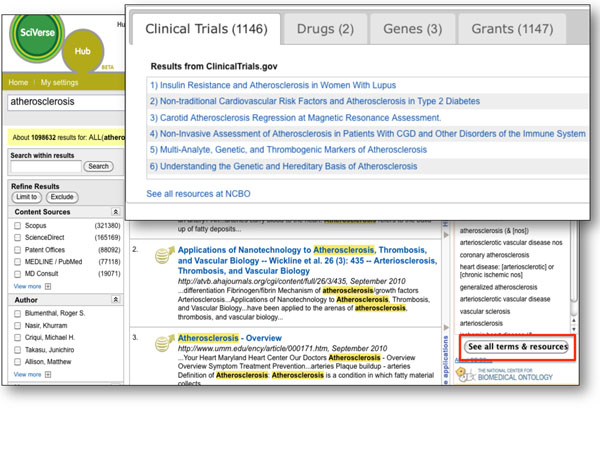
**Ontology Driven Semantic Search** Search results for the term “atherosclerosis” are highlighted in the abstract results. The *Annotator* Web service recognizes ontology terms in these abstracts and these terms are displayed in the ODiSSea application window. Clicking on the “See all terms & resources” button displays a pop-up window with results from searching the Resource Index.

The ***Ontology Driven Semantic Search*** SciVerse application uses both the *Annotator* and *Resource Index* Web services (Figure 8). Ontology terms within abstracts in SciVerse are first identified using the *Annotator* Web service and then these terms are used as input to perform a search of ClinicalTrials.gov, DrugBank, OMIM, Research Crossroads via the *Resource Index* Web services to link the abstracts to information about clinical trials, drugs, genes, and grants.

## Summary

The suite of NCBO Web services power a variety of semantically aware software applications (see additional file 1). The Web services are used in various combinations to enable workflows for ontology development, data annotation, and data analysis. Future work will include expansion of the Web services to enhance selection of terms by ontology sub-setting, to build lexicons for use with the *Annotator* Web service, and for ontology enrichment analysis.

## Competing interests

No competing interests.

## Authors' contributions

PLW drafted the manuscript.

## Authors' information

PLW is the Outreach Coordinator for the National Center for Biomedical Ontology.

## Supplementary Material

Additional file 1 **Software applications using NCBO Technology** A number of software applications that are using NCBO technology is listed. Click here for file

Additional file 2 Click here for file

## Declarations

The publication costs for this article were funded by the corresponding author's institution.

This article has been published as part of *Journal of Biomedical Semantics* Volume 4 Supplement 1, 2013: Proceedings of the Bio-Ontologies Special Interest Group 2012. The full contents of the supplement are available online at http://www.jbiomedsem.com/supplements/4/S1
