# Supplementary material for: NCBO Technology: Powering semantically aware applications
Source: J Biomed Semantics. 2013 Apr 15;4(Suppl 1):S8. doi: 10.1186/2041-1480-4-S1-S8 (PMC3633000; doi:10.1186/2041-1480-4-S1-S8)
Supplement: Additional file 1 — Software applications using NCBO Technology A number of software applications that are using NCBO technology is listed. [file 2041-1480-4-S1-S8-S1.docx]

Software applications using NCBO Technology.

| **Software** | **Description** | **Technology** |
| --- | --- | --- |
| ALEX | Provide learning management system that is a central resource for online medical education content and computer-based learning activities. https://alex.med.nyu.edu/portal | W |
| aTag Generator | Create snippets of HTML that capture the information that is most important to a user in a machine-readable, interlinked format. http://hcls.deri.org/atag/generator/ | W |
| BioDAG Builder | Input a list of ontology identifiers or upload a file containing terms. Then output a custom ontology graph for these specific terms in OWL and OBO format. <http://viti.gene.le.ac.uk/tree/index.php> | W |
| BioLit | Extract database identifiers and rich meta-data from open access articles in the life sciences and integrate that information with existing biological databases. http://biolit.ucsd.edu/doc/ | O |
| BioPortal Reference Plug-in | Insert ontology class references into documents. http://protegewiki.stanford.edu/wiki/BioPortal_Reference_ Plugin | O |
| BioPortal Import Plug-in | Import classes from ontologies, allowing users to choose entire trees of classes with a desired depth and to choose which properties to import for each class. http://protegewiki.stanford.edu/wiki/BioPortal_Import_ Plugin | O |
| BioScholar | Support experimental biomedical scientists, allowing a single scientific worker (at the level of a graduate student or postdoctoral worker) to design, construct, and manage a shared knowledge repository for a research group derived on a local store of PDF files. https://wiki.birncommunity.org/display/NEWBIRNCC/ BioScholar | O |
| Biositemaps Editor | Generate Biositemap description of a user resource. http://biositemaps.ncbcs.org/ | O |
| Case-Based Reasoning System | Acquire and manage knowledge repositories. http://vphenodbs-dev.rnet.missouri.edu/~hc79b/KITE/ index.php | W |
| cgMDR | Annotate data elements within the metadata registry being developed by CancerGrid.  http://goo.gl/dvsgM | O |
| CISBIC Data Management | Share, integrate, and archive data from various sources so that computational and statistical analyses can propose new hypotheses for experimental verification. http://www3.imperial.ac.uk/cisbic/corefacilities/ datamanagement | O |
| Corona | Microarray annotation tool. Internal curation tool. | O, W |
| Domeo | An extensible web application enabling users to visually and efficiently create and share ontology-based stand-alone annotations. The tool supports manual, fully automated, and semi-automated annotation with complete provenance records, as well as personal or community annotation with access authorization and control. <http://annotationframework.org/> | A |
| DXBrain Project | Create distributed data integration system for the Human Brain Project data network. http://xbrain.biostr.washington.edu:8080/ dxbrain-gui/index.jsp | W |
| eleMAP | Allow researchers to harmonize local phenotype data dictionaries to existing metadata and terminology standards such as the caDSR (Cancer Data Standards Registry and Repository), NCIT (NCI Thesaurus) and SNOMED-CT (Systematized Nomenclature of Medicine-Clinical Terms). https://victr.vanderbilt.edu/eleMAP/ | O |
| GeneWiki | Provide informal place to collect information on human genes and proteins. http://en.wikipedia.org/wiki/Portal:Gene_Wiki | A |
| GMiner | Allow search of rat microarray experiments. http://gminer.mcw.edu/ | W |
| GWAS Central | A centralized compilation of summary level findings from genetic association studies, both large and small. We actively gather datasets from public domain projects, and encourage direct data submission from the community. <https://www.gwascentral.org/index> | W |
| iCAT | Provide tools for ICD-11 collaborative authoring. http://sites.google.com/site/icd11revision/home/icat | O |
| ISAcreator | Allow experimentalists to report, edit experimental metadata, and ultimately validate their data files. http://isatab.sourceforge.net/isacreator.html | O, A |
| Jinx | Annotate brain images. http://ncmir.ucsd.edu/downloads/jinx.shtm | O |
| Knowledge Egg | Search across resources.  http://www.kunnskapsegget.no | W |
| MAVIR | Develop web mining and document classification techniques. http://www.mavir.net/groups/uem | A |
| mEducator | Implement and critically evaluate existing standards and reference models in the field of e-learning to enable specialized state-of-the-art medical educational content to be discovered, retrieved, shared and re-used http://meducator.med.auth.gr | W |
| MeRy-B | MeRy-B is a plant metabolomics knowledgebase allowing the storage and visualization of metabolic profiles from plants. <http://services.cbib.u-bordeaux2.fr/MERYB/about/home.php> | W |
| Microsoft Word Addin for Ontology Recognition | Enable annotation of Word documents based on terms that appear in ontologies.  http://ucsdbiolit.codeplex.com/ | O |
| MG-RAST | Provide automated analysis platform for metagenomes that allows quantitative insights into microbial populations based on sequence data.  http://metagenomics.anl.gov/ | W |
| Modularize | Extracts subsets of ontologies, including all axioms logically implied to be necessary and sufficient for complete reasoning over the signature. <http://sswap.info/modularize> | O |
| NEMO Toolkit | Provide tools for EEG/ERP and MEG data decomposition, ontology-based mark-up, annotation, and labeling of patterns in EEG and MEG data.  http://nemo.nic.uoregon.edu/wiki/ NEMO_ERP_Analysis_Toolkit | O |
| NCBO-Galaxy | NCBO-Galaxy provides graphical interfaces for the NCBO Web services available at BioPortal, to access and exploit biomedical ontologies as part of Galaxy workflows. <http://toolshed.g2.bx.psu.edu/> | O, A, RI |
| NIFSTD | Annotation of resources in NIF, an inventory of Web-based neuroscience resources. <http://www.neuinfo.org/> | A |
| NMC Data Support Platform | Collect, store, and share biological study data. <http://ci.nmcdsp.org/> | W |
| Nutritional Phenotype Database | Help biologists to interpret the results of biology studies that involve multiple 'omics' techniques.  http://www.dbnp.org | W |
| ODIE | Provide an open-source, extensible toolkit for ontology annotation and enrichment from clinical text. http://www.bioontology.org/ODIE-project | A |
| ODiSSea | Expand queries with standard ontologies and search public data resources for additional information on clinical trials, genes, drugs, and funding.  http://www.hub.sciverse.com | O, A, RI |
| OntoCat | Interact with a wide array of ontology resources http://www.ontocat.org | O |
| OntoFinder  OntoFactory | Enables ontology term re-use through search and extract of terms of interest. <http://ontofinder.dbcls.jp/> | O |
| OntoGrator | Ontology-based search of ClinicalTrials.gov. <http://www.ontogrator.org/> | A |
| Ontological Discovery Environment | Integrate phenotype centered gene sets across species, tissue, and experimental platform. http://ontologicaldiscovery.org | A |
| openMDR | Enable smaller groups or institutions to easily create local metadata registries and curate semantic metadata. http://citih.osumc.edu/projects/project&r=1032 | O |
| Oryzabase | Create a comprehensive rice science database. http://www.shigen.nig.ac.jp/rice/oryzabase/top/top.jsp | W |
| QIIME | Comparison and analysis of microbial communities, primarily based on high-throughput amplicon sequencing data (such as SSU rRNA) <http://qiime.org/index.html> | W |
| RadLex Tree Browser | Provide customized view of RadLex by the Radiological Society of North America.  http://www.radlex.org | O, W |
| RadSpeech | Provide semantic speech dialogue system for radiologists. http://digitaleveredelung.dfki.de/MEDICO-Playground/ term2.html | W |
| REDfly | Provide curated collection of known Drosophila transcriptional cis-regulatory modules (CRMs) and transcription factor binding sites (TFBSs). http://redfly.ccr.buffalo.edu | W |
| **Resource of Asian Primary Immunodeficiency Diseases** | A web-based informatics platform, which enables PID experts to easily mine collected genomic, transcriptomic, and proteomic data of PID causing genes. <http://rapid.rcai.riken.jp/RAPID> | O, W |
| RGXpress | Online manuscript and peer review system. <http://rgxpress.rsna.org/index.cfm> | W |
| RightField | Add ontology term selection to Excel spreadsheets. http://www.sysmo-db.org/rightfield | O |
| S3DB | Represent information on the Semantic Web without the rigidness of relational/XML schema while avoiding the "spaghetti" of unconstrained RDF stores. https://sites.google.com/a/s3db.org/s3db | O |
| SEEK | Provide asset management tool that allows consortium members to register assets and search for any assets for which they have access rights.  http://www.sysmo-db.org/seek | O |
| Semantic Medical Image Annotation | Search for radiology images based on anatomical location, disease classification, or radiology finding. http://kithira.biosim.ntua.gr/semia | O, W |
| Sentient Knowledge Explorer | Integrate data from virtually any source into coherent, unified knowledge bases.  http://www.io-informatics.com/ | O |
| SimTK | Provide technologies for building applications that employ physics-based simulations of biological structures. https://simtk.org/home/simtk | W |
| STRIDE | Create a standards-based informatics platform supporting clinical and translational research. https://clinicalinformatics.stanford.edu/research/stride.html | A |
| TRIAD | Create a scalable, secure, and knowledge-anchored data- sharing environment.  http://triadcommunity.org | O |
| Tripod | Create a user-friendly chemical genomics browser. http://tripod.nih.gov | A |
| Zooma | Support discovering optimal ontology mappings and automatically map text values to ontology terms. http://zooma.sourceforge.net | O |

The technology categories are Ontology (O), Annotator (A), Resource Index (RI), and Widgets (W).
