# Supplementary material for: NCBO Technology: Powering semantically aware applications
Source: J Biomed Semantics. 2013 Apr 15;4(Suppl 1):S8. doi: 10.1186/2041-1480-4-S1-S8 (PMC3633000; doi:10.1186/2041-1480-4-S1-S8)
Supplement: Additional file 2 [file 2041-1480-4-S1-S8-S2.pdf]

Software applications using NCBO Technology.

Software	Description	Technology
ALEX	Provide learning management system that is a central resource for online medical education content and computer-based learning activities. https://alex.med.nyu.edu/portal	W
aTag Generator	Create snippets of HTML that capture the information that is most important to a user in a machine-readable, interlinked format. http://hcls.deri.org/atag/generator/	W
BioDAG Builder	Input a list of ontology identifiers or upload a file containing terms. Then output a custom ontology graph for these specific terms in OWL and OBO format. http://viti.gene.le.ac.uk/tree/index.php	W
BioLit	Extract database identifiers and rich meta-data from open access articles in the life sciences and integrate that information with existing biological databases. http://biolit.ucsd.edu/doc/	O
BioPortal Reference Plug-in	Insert ontology class references into documents. http://protegewiki.stanford.edu/wiki/BioPortal_Reference_Plugin	O
BioPortal Import Plug-in	Import classes from ontologies, allowing users to choose entire trees of classes with a desired depth and to choose which properties to import for each class. http://protegewiki.stanford.edu/wiki/BioPortal_Import_Plugin	O
BioScholar	Support experimental biomedical scientists, allowing a single scientific worker (at the level of a graduate student or postdoctoral worker) to design, construct, and manage a shared knowledge repository for a research group derived on a local store of PDF files. https://wiki.birncommunity.org/display/NEWBIRNCC/BioScholar	O
Biositemaps Editor	Generate Biositemap description of a user resource. http://biositemaps.ncbcs.org/	O
Case-Based Reasoning System	Acquire and manage knowledge repositories. http://vphenodbs-dev.rnet.missouri.edu/~hc79b/KITE/index.php	W
cgMDR	Annotate data elements within the metadata registry being developed by CancerGrid. http://goo.gl/dvsgM	O
CISBIC Data Management	Share, integrate, and archive data from various sources so that computational and statistical analyses can propose new hypotheses for experimental verification. http://www3.imperial.ac.uk/cisbic/corefacilities/datamanagement	O

Software	Description	Technology
Corona	Microarray annotation tool. Internal curation tool.	O, W
Domeo	An extensible web application enabling users to visually and efficiently create and share ontology-based stand-alone annotations. The tool supports manual, fully automated, and semi-automated annotation with complete provenance records, as well as personal or community annotation with access authorization and control. http://annotationframework.org/	A
DXBrain Project	Create distributed data integration system for the Human Brain Project data network. http://xbrain.biostr.washington.edu:8080/ dxbrain-gui/index.jsp	W
eleMAP	Allow researchers to harmonize local phenotype data dictionaries to existing metadata and terminology standards such as the caDSR (Cancer Data Standards Registry and Repository), NCIT (NCI Thesaurus) and SNOMED-CT (Systematized Nomenclature of Medicine-Clinical Terms). https://victr.vanderbilt.edu/eleMAP/	O
GeneWiki	Provide informal place to collect information on human genes and proteins. http://en.wikipedia.org/wiki/Portal:Gene_Wiki	A
GMiner	Allow search of rat microarray experiments. http://gminer.mcw.edu/	W
GWAS Central	A centralized compilation of summary level findings from genetic association studies, both large and small. We actively gather datasets from public domain projects, and encourage direct data submission from the community. https://www.gwascentral.org/index	W
iCAT	Provide tools for ICD-11 collaborative authoring. http://sites.google.com/site/icd11revision/home/icat	O
ISAcreator	Allow experimentalists to report, edit experimental metadata, and ultimately validate their data files. http://isatab.sourceforge.net/isacreator.html	O, A
Jinx	Annotate brain images. http://ncmir.ucsd.edu/downloads/jinx.shtm	O
Knowledge Egg	Search across resources. http://www.kunnskapsegget.no	W
MAVIR	Develop web mining and document classification techniques. http://www.mavir.net/groups/uem	A
mEducator	Implement and critically evaluate existing standards and reference models in the field of e-learning to enable specialized state-of-the-art medical educational content to be discovered, retrieved, shared and re-used http://meducator.med.auth.gr	W

Software	Description	Technology
MeRy-B	MeRy-B is a plant metabolomics knowledgebase allowing the storage and visualization of metabolic profiles from plants. http://services.cbib.u-bordeaux2.fr/MERYB/about/home.php	W
Microsoft Word Addin for Ontology Recognition	Enable annotation of Word documents based on terms that appear in ontologies. http://ucsdbiolit.codeplex.com/	O
MG-RAST	Provide automated analysis platform for metagenomes that allows quantitative insights into microbial populations based on sequence data. http://metagenomics.anl.gov/	W
Modularize	Extracts subsets of ontologies, including all axioms logically implied to be necessary and sufficient for complete reasoning over the signature. http://sswap.info/modularize	O
NEMO Toolkit	Provide tools for EEG/ERP and MEG data decomposition, ontology-based mark-up, annotation, and labeling of patterns in EEG and MEG data. http://nemo.nic.uoregon.edu/wiki/ NEMO_ERP_Analysis_Toolkit	O
NCBO-Galaxy	NCBO-Galaxy provides graphical interfaces for the NCBO Web services available at BioPortal, to access and exploit biomedical ontologies as part of Galaxy workflows. http://toolshed.g2.bx.psu.edu/	O, A, RI
NIFSTD	Annotation of resources in NIF, an inventory of Web-based neuroscience resources. http://www.neuinfo.org/	A
NMC Data Support Platform	Collect, store, and share biological study data. http://ci.nmcdsp.org/	W
Nutritional Phenotype Database	Help biologists to interpret the results of biology studies that involve multiple 'omics' techniques. http://www.dbnp.org	W
ODIE	Provide an open-source, extensible toolkit for ontology annotation and enrichment from clinical text. http://www.bioontology.org/ODIE-project	A
ODiSSea	Expand queries with standard ontologies and search public data resources for additional information on clinical trials, genes, drugs, and funding. http://www.hub.sciverse.com	O, A, RI
OntoCat	Interact with a wide array of ontology resources http://www.ontocat.org	O
OntoFinder OntoFactory	Enables ontology term re-use through search and extract of terms of interest. http://ontofinder.dbcls.jp/	O
OntoGrator	Ontology-based search of ClinicalTrials.gov. http://www.ontogratorg.org/	A

Software	Description	Technology
Ontological Discovery Environment	Integrate phenotype centered gene sets across species, tissue, and experimental platform. http://ontologicaldiscovery.org	A
openMDR	Enable smaller groups or institutions to easily create local metadata registries and curate semantic metadata. http://citih.osumc.edu/projects/project&r=1032	O
Oryzabase	Create a comprehensive rice science database. http://www.shigen.nig.ac.jp/rice/oryzabase/top/top.jsp	W
QIIME	Comparison and analysis of microbial communities, primarily based on high-throughput amplicon sequencing data (such as SSU rRNA) http://qiime.org/index.html	W
RadLex Tree Browser	Provide customized view of RadLex by the Radiological Society of North America. http://www.radlex.org	O, W
RadSpeech	Provide semantic speech dialogue system for radiologists. http://digitaleveredelung.dfki.de/MEDICO-Playground/ term2.html	W
REDfly	Provide curated collection of known Drosophila transcriptional cis-regulatory modules (CRMs) and transcription factor binding sites (TFBSs). http://redfly.ccr.buffalo.edu	W
Resource of Asian Primary Immunodeficiency Diseases	A web-based informatics platform, which enables PID experts to easily mine collected genomic, transcriptomic, and proteomic data of PID causing genes. http://rapid.rcai.riken.jp/RAPID	O, W
RGXpress	Online manuscript and peer review system. http://rgxpress.rsna.org/index.cfm	W
RightField	Add ontology term selection to Excel spreadsheets. http://www.sysmo-db.org/rightfield	O
S3DB	Represent information on the Semantic Web without the rigidness of relational/XML schema while avoiding the "spaghetti" of unconstrained RDF stores. https://sites.google.com/a/s3db.org/s3db	O
SEEK	Provide asset management tool that allows consortium members to register assets and search for any assets for which they have access rights. http://www.sysmo-db.org/seek	O
Semantic Medical Image Annotation	Search for radiology images based on anatomical location, disease classification, or radiology finding. http://kithira.biosim.ntua.gr/semia	O, W
Sentient Knowledge Explorer	Integrate data from virtually any source into coherent, unified knowledge bases. http://www.io-informatics.com/	O

Software	Description	Technology
SimTK	Provide technologies for building applications that employ physics-based simulations of biological structures. https://simtk.org/home/simtk	W
STRIDE	Create a standards-based informatics platform supporting clinical and translational research. https://clinicalinformatics.stanford.edu/research/stride.html	A
TRIAD	Create a scalable, secure, and knowledge-anchored data-sharing environment. http://triadcommunity.org	O
Tripod	Create a user-friendly chemical genomics browser. http://tripod.nih.gov	A
Zooma	Support discovering optimal ontology mappings and automatically map text values to ontology terms. http://zooma.sourceforge.net	O

The technology categories are Ontology (O), Annotator (A), Resource Index (RI), and Widgets (W).
